# iHAP – integrated haplotype analysis pipeline for characterizing the haplotype structure of genes

**DOI:** 10.1186/1471-2105-7-525

**Published:** 2006-12-01

**Authors:** Chun Meng Song, Boon Huat Yeo, Erwin Tantoso, Yuchen Yang, Yun Ping Lim, Kuo-Bin Li, Gunaretnam Rajagopal

**Affiliations:** 1 Bioinformatics Institute, 30 Biopolis Street, #07-01, 138671, Singapore; 2 Institute of Molecular Cell and Biology, 61 Biopolis Drive (Proteos), 138673, Singapore; 3 Bioinformatics Center, National Yang-Ming University, Taipei, 112, Taiwan

## Abstract

**Background:**

The advent of genotype data from large-scale efforts that catalog the genetic variants of different populations have given rise to new avenues for multifactorial disease association studies. Recent work shows that genotype data from the International HapMap Project have a high degree of transferability to the wider population. This implies that the design of genotyping studies on local populations may be facilitated through inferences drawn from information contained in HapMap populations.

**Results:**

To facilitate analysis of HapMap data for characterizing the haplotype structure of genes or any chromosomal regions, we have developed an integrated web-based resource, iHAP. In addition to incorporating genotype and haplotype data from the International HapMap Project and gene information from the UCSC Genome Browser Database, iHAP also provides capabilities for inferring haplotype blocks and selecting tag SNPs that are representative of haplotype patterns. These include block partitioning algorithms, block definitions, tag SNP definitions, as well as SNPs to be "force included" as tags. Based on the parameters defined at the input stage, iHAP performs on-the-fly analysis and displays the result graphically as a webpage. To facilitate analysis, intermediate and final result files can be downloaded.

**Conclusion:**

The iHAP resource, available at , provides a convenient yet flexible approach for the user community to analyze HapMap data and identify candidate targets for genotyping studies.

## Background

The identification of Single Nucleotide Polymorphisms (SNPs) that contribute to complex diseases has made them the preferred choice for diagnostics and therapeutics studies. For instance, the methylenetetrahydrofolate reductase (MTHFR) C677T polymorphism (dbSNP: rs1801133) has been reported to be associated with gastric cancer in the Chinese population [1]. To uncover novel markers that may be associated to a disease, genotyping studies are conducted to determine the genetic variations between diseased and healthy subjects, allowing for further functional characterization that could lead to therapeutic applications. While it may not be sufficient coverage just to genotype only these specific disease-related SNPs, it is costly to genotype all available SNPs from a large sample of individuals. As such, by genotyping only a subset (also known as the tag SNPs [2]), which may include the disease associated SNPs, the cost and effort involved in association studies can be effectively reduced with minimal compromise to the power of such studies.

In the absence of comprehensive genotype data from local populations, genotyping studies can be designed using data from the International HapMap Project [3]. Recent studies show that, despite differences in the fine details of linkage disequilibrium (LD) patterns between populations [4], tag SNPs selected from one HapMap population can be used to characterize other populations reasonably well [5-7]. These findings indicate that HapMap data is currently the most ideal freely-available dataset for tag SNP selection and association studies.

Currently, there exist several tools to facilitate haplotype block inference and tag SNP selection. For example, the International HapMap Project website [8] not only provides bulk download of genotype and frequency data from the International HapMap Project, but also interactive access to visualize the distribution of SNPs for any genomic region of interest. Haploview [9] is a standalone application that performs LD and haplotype block analysis on publicly available or user supplied genotype data. htSNPer1.0 [10] and HaploBlock [11] can also be used to analyze genotype data supplied by users. HaploBlockFinder [12] is a web-based tool that allows for the inference of haplotype blocks and tag SNP selection from genotype data uploaded by users. The Genome Variation Server (GVS) [13] and PupaSuite [14] are other tools with several online analysis utilities for accessing human genotype data. More recently, TAMAL [15] was developed adopting a pre-processing strategy to facilitate the selection of potential genotyping targets.

As a web-based tool, the iHAP resource complements the capabilities of existing tools for analyzing haplotype structures and selecting tag SNPs using data from HapMap. While most tools deploy limited algorithms for selecting tag SNPs, iHAP provides a wider selection of algorithms and parameter settings for both haplotype block partitioning as well as tag SNP definitions. This is achieved through the use of HapBlock [16] as iHAP's backend haplotype analysis tool. The HapMap Project website and Haploview select tag SNPs using Tagger [17], which is based on the pairwise LD *r*^2 ^[2] method. In addition to *r*^2 ^methods, iHAP incorporates tag SNP definitions including common haplotypes [18], haplotype diversity [19], haplotype entropy [20] and haplotype determination coefficient [21]. This provides users with fully customizable options for on-the-fly analysis as opposed to pre-processed results provided by TAMAL. iHAP also highlights SNPs found in coding regions so potentially significant SNPs may be "force included" as tag SNPs at users' discretion, a feature unavailable in GVS and TAMAL. Being integrated with our local repositories of genotype and gene data from HapMap and the UCSC Genome Browser Database [22] respectively, iHAP relieves users of the hassle of having to locate and download genotype data as is the case with Haploview. Furthermore, iHAP generates result pages that graphically depict the haplotype structures, including blocks, haplotype patterns and tag SNPs, alongside the exons and introns of genes found within the chromosomal region. Alternative sets of inferred tag SNPs are also presented with the respective scores. The key differences between iHAP and other similar tools are highlighted in Table 1.

**Table 1:** Comparison of iHAP vs other similar tools

	**iHAP**	**GVS**	**TAMAL**	**PupaSuite**	**htSNPer1.0**
Integration with HapMap data?	√	√	√	√	X
Integration of gene information?	√	√	√	√	X
Accept user's genotype data?	X	√	X	√	√
Web-based?	√	√	√	√	X
Force tag SNP selection?	√	X	X	X	X
Variety of block partitioning algorithms?	√	X	X	X	√
Variety of tag SNP definitions?	√	X	√	X	√
Graphical display of blocks and genes along chromosome?	√	X	X	X	X
Real-time analysis?	√	√	X	√	√

## Implementation

**Figure 1:**
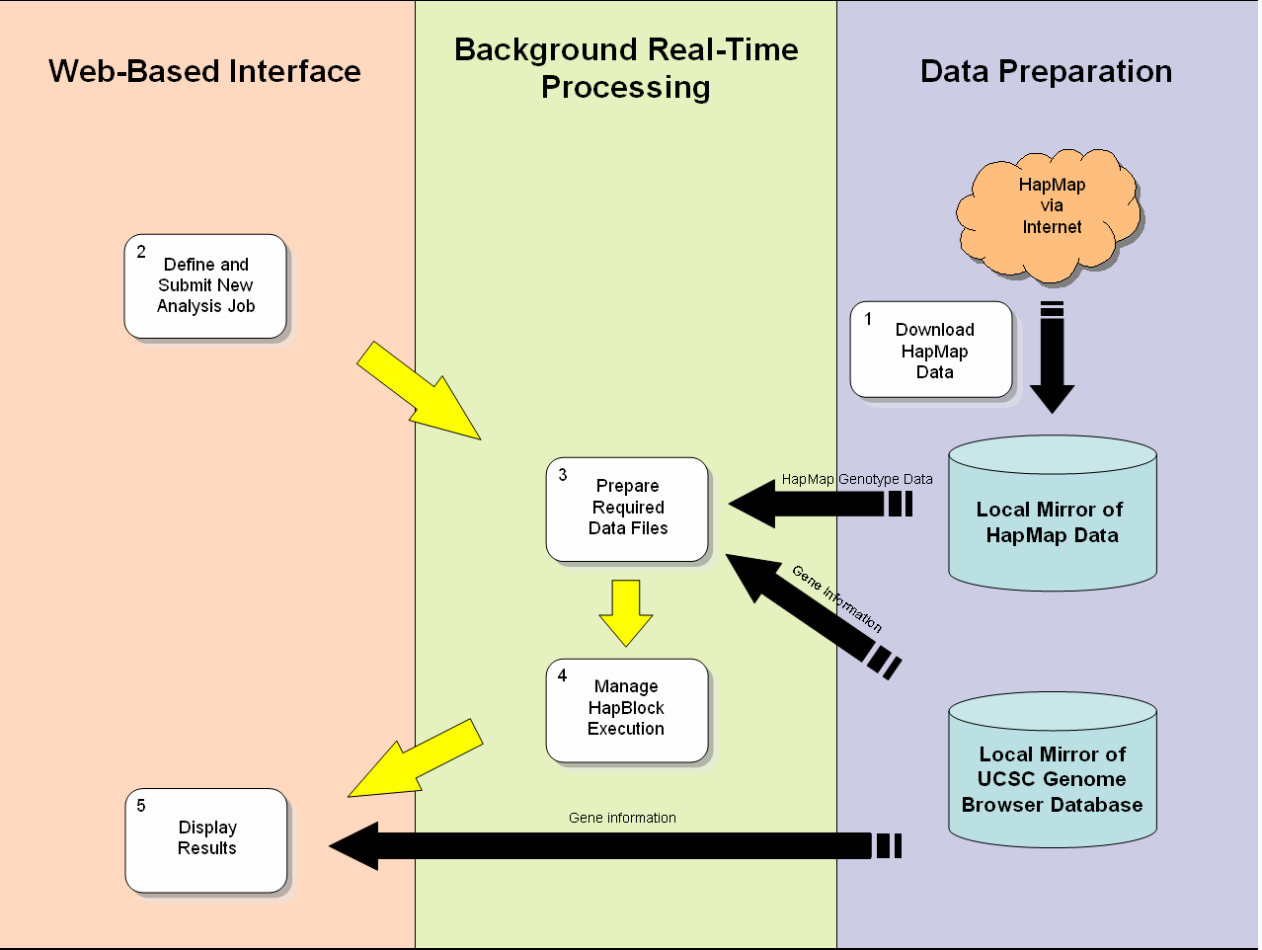
**Overall schematic of iHAP**. The iHAP resource may be conceptualized as having three components. The first involves batch-based data preparation while the second and the third are for real time analyses. Users submit jobs to iHAP via the web-based interface and each job is then processed in the background. Upon completion, results are returned to the users via the web-based interface.

The iHAP resource was written in the PHP 5.1.4 scripting language with the GD library of image functions. Using a backend MySQL 4.1.14 relational database, this resource is currently deployed on a Solaris environment with Apache HTTP Server 2.0.58 running on a Sun Fire V240 Server. An overall schematic architecture of iHAP is shown in Figure 1.

### Choice of backend haplotype analysis tool

Apart from HapBlock, other tools including HaploBlock and HaploBlockFinder were also considered and evaluated for suitability as iHAP's backend haplotype analysis tool. Eventually, HapBlock was preferred over these alternatives because it offers a wider selection of haplotype block definitions and tag SNP selection algorithms. HapBlock is also capable of accommodating the option for "forcing" specific SNPs to be selected as tags, which is helpful if one wants to include prior information into the analysis.

### Local data repositories

Essential to the execution of the iHAP resource are two data repositories. The first is a database created for storing HapMap data (HapMap Public Release #21, Jul 2006, on NCBI Build 35 assembly). This database adopts a schema that was designed to support efficient queries for genotype and haplotype data for any population and genomic regions of interest. Genotype data of the four HapMap human populations, namely CEPH (Utah Residents with Northern and Western European Ancestry) (CEU), Han Chinese in Beijing, China (CHB), Japanese in Tokyo, Japan (JPT), Yoruba in Ibadan, Nigeria (YRI), was subsequently downloaded from the International HapMap Project website [8] and populated into the local database.

The other resource is the local mirror of the UCSC Genome Browser Database. To ensure that the SNP positions obtained from HapMap are consistent with the gene locations from the UCSC Genome Browser Database, the hg17 assembly is used. The human reference sequence in this assembly is based on NCBI build 35. This resource is used for determining the chromosomal locations of genes, including the positions of their respective introns and exons.

### Job execution and management

Based on the settings supplied by users, iHAP generates the necessary input files in the format required by HapBlock and triggers its execution as a background job. Depending on the nature of individual haplotype analysis jobs, the execution time could vary from seconds to hours. In addition, the storage requirements for each job also vary according to the availability of genotype data for the selected chromosomal region. Therefore, it is necessary to optimize job scheduling to present users with a logical and coherent interface without compromising server performance.

To address this issue, a job manager module was devised. This module not only initiates each HapBlock execution as a background job, but also monitors the execution process through periodic polls. With this module, the progress of each job can be tracked so users may be updated with the current status of their jobs via the web-based interface. An email alert mechanism is also in place to inform users upon completion of their analysis jobs. To keep storage requirements in check, a script that automatically cleans up redundant files belonging to old jobs is also executed periodically.

### Result display

As individual jobs are completed, the job manager module extracts information pertaining to haplotype blocks and tag SNPs to an intermediate format. Alternative sets of results are collated along with their respective scores while exon and intron information of genes found within the chromosomal region of interest is obtained from the local mirror of the UCSC Genome Browser Database. Such information is then combined along with additional details such as SNP names and locations in the dynamically generated image that illustrates the haplotype structure graphically. Intermediate files relating to individual jobs are finally archived in ZIP files which can be downloaded conveniently.

## Results and discussion

Based on the submitted gene name, the iHAP resource determines the chromosomal region of interest using the UCSC Genome Browser Database. The setup of the analysis job is then defined according to parameters such as HapMap population, allele frequency threshold, block definitions, tag SNP definitions, permutation test settings, as well as SNPs to be "force included" as tags. Snippets of help for each parameter are ergonomically positioned to facilitate the configuration of each job.

The necessary files required by HapBlock are then generated by iHAP. These include the parameter, genotype or haplotype data, SNP names, SNP position lookup, and "forced tag SNP" files. iHAP then invokes the execution of HapBlock as a background job and monitors its progress through periodic polls so as to keep users updated on their job progression. Upon completion of the analysis, results are converted to dynamically generated images for display as a webpage.

**Figure 2:**
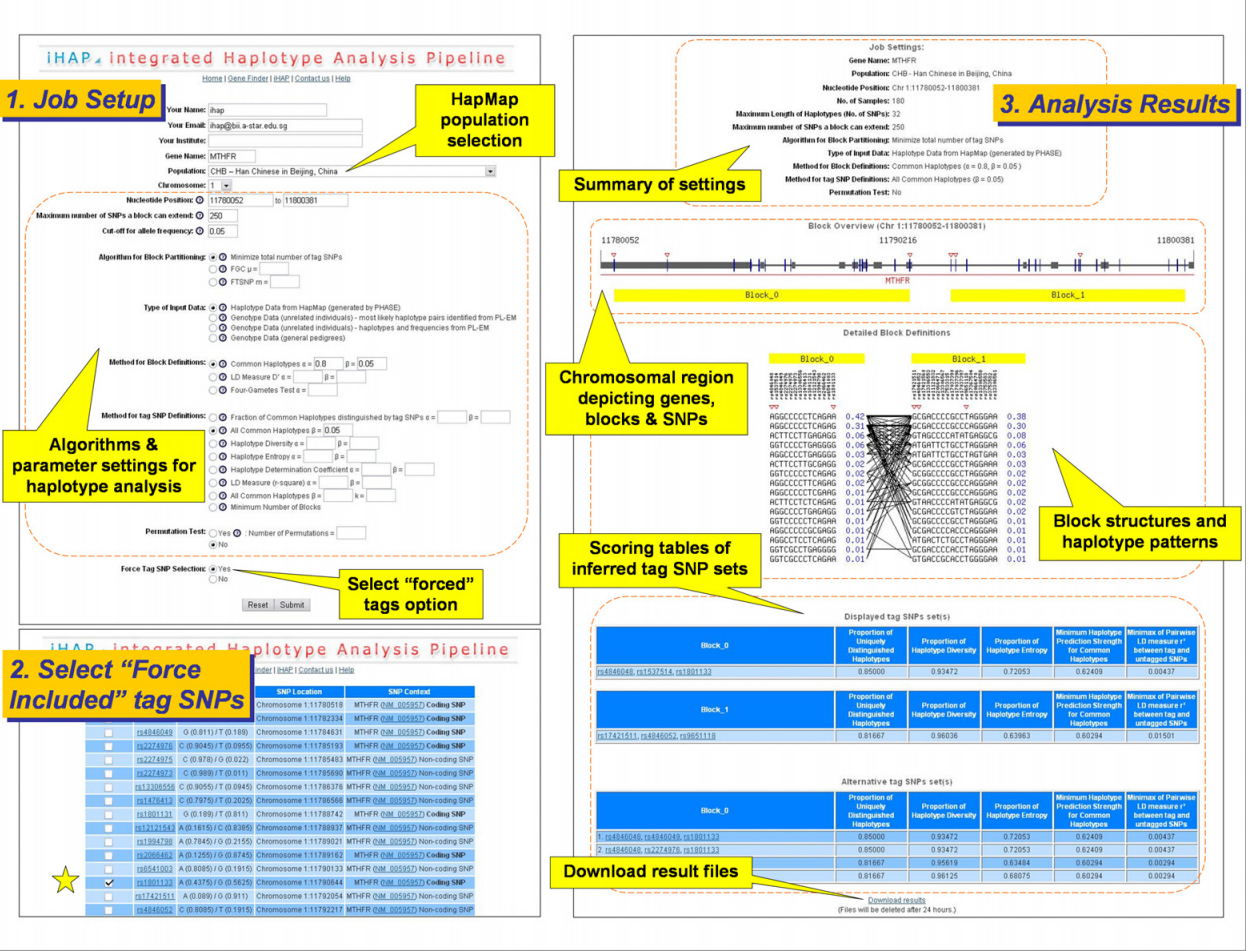
**Typical workflow of iHAP**. The iHAP resource was used to analyze the MTHFR gene with gastric cancer related SNP (rs1801133) "force included" as tag.

The results page first provides a summary of the settings used for the analysis. A graphical representation of the genomic region is displayed with the locations of genes and their respective intronic and exonic regions illustrated as grey boxes, and inferred blocks as yellow rectangles. SNP locations are marked as blue vertical lines with those selected as tags augmented with red triangles. The next section depicts the structure of each block, including the dbSNP identifiers and the haplotype patterns along with their respective frequencies. The scores of the displayed and alternative tag SNP sets for each block are tabulated according to various criteria in the following section. Finally, an archive (ZIP) file containing all the files generated in the analysis can be downloaded via a link on the results page. A typical analysis flow is exemplified in Figure 2.

## Conclusion

The iHAP application provides a one-stop resource for inferring haplotype blocks and selecting tag SNPs from HapMap data. Apart from providing a wider selection of algorithms and integrating genotype data with gene information, iHAP also offers greater flexibilities by allowing users to "force include" specific SNPs as tags. Additionally, iHAP displays the results obtained graphically for intuitive interpretation and includes alternative sets of tag SNPs attained. In essence, iHAP is a practical tool that can be used to analyze HapMap data for the selection of candidate targets in genotyping studies.

## Availability and requirements

**Project name: **iHAP (integrated haplotype analysis pipeline)

**Project home page: **

**Operating system: **Solaris (or any other OS that supports Apache, MySQL and PHP)

**Programming language: **PHP (with GD library)

**Other requirements: **MySQL, Apache HTTP Server

**License: **none

**Any restrictions to use by non-academics: **On request and citation

## Authors' contributions

The iHAP resource was conceptualized and developed by CMS, BHY, ET and YY. While CMS and BHY prepared the data and wrote the programs, ET and YY evaluated suitable backend haplotype analysis tools. KBL and GR supervised the project and provided guidance while YPL facilitated the process. CMS, BHY, ET and YY drafted the manuscript and all authors have read and approved the final manuscript.
